# The role of a low glomerular density and being overweight in the etiology of proteinuria in CKD patients without known glomerular diseases

**DOI:** 10.1007/s10157-014-0940-y

**Published:** 2014-02-11

**Authors:** Hidekazu Okamoto, Tetsuya Kawamura, Hideo Okonogi, Nobuo Tsuboi, Yoichi Miyazaki, Takashi Yokoo

**Affiliations:** Division of Kidney and Hypertension, Department of Internal Medicine, The Jikei University School of Medicine, 3-25-8 Nishi-shinbashi, Minato-ku, Tokyo, 105-8461 Japan

**Keywords:** Chronic kidney disease, Glomerular hypertrophy, Proteinuria, Overweight, Obesity, Body mass index, Renal biopsy, Glomerular density

## Abstract

**Background:**

Among the proteinuric patients with chronic kidney disease (CKD) who undergo a renal biopsy, we sometimes encounter those who cannot be classified as having a known primary or secondary glomerular disease. The pathogenesis and pathophysiology of these CKD patients have not been sufficiently elucidated.

**Methods:**

We recruited 34 proteinuric patients without known glomerular diseases. The glomerular volumes (GV) of the biopsy specimens from those patients were determined by a morphometric analysis. Glomerular hypertrophy (GH) was defined as having more than 3.6 × 10^6^ μm^3^. The patients were divided in two groups: those with GH (Group 1) and those without GH (Group 2). We compared the clinical and pathological parameters between Group 1 and Group 2, and among the three groups of patients: non-obese, overweight and obese group.

**Results:**

The patients with Group 1 had significantly higher values for the proportion of males, the body mass index (BMI), uric acid and significantly lower values for the glomerular density (GD). Of note, a multivariate regression analysis revealed that sex, the BMI and GD were significant factors correlated with the mean GV. The values for the mean GV were significantly higher in the overweight and obese groups as compared to the non-obese group, and the values for the GD were significantly lower in the obese group than in the non-obese group.

**Conclusions:**

We identified a subgroup of patients who were characterized as having a high BMI and GV, and a low GD among the proteinuric CKD patients without known glomerular diseases.

## Introduction

Chronic kidney disease (CKD) is a worldwide public health problem [1, 2]. It has been reported that the prevalence of CKD is 13 % in the United States [3], 10–13 % in European countries [4, 5], 13 % in Japan [6] and 12–13 % in China [7]. Of all CKD patients, those with proteinuria have been shown to have a higher risk of developing cardiovascular disease (CVD) [8], as well as end-stage renal disease (ESRD) [9]. Although a renal biopsy is a useful diagnostic procedure to elucidate the pathogenesis in proteinuric patients, we sometimes encounter those who do not fit the diagnostic criteria for any known primary or secondary glomerular diseases. The pathogenesis and pathophysiology of these CKD patients have not been sufficiently elucidated.

On the other hand, previous experimental and clinical studies demonstrated that glomerular hypertrophy (GH) plays an important role in the progression of glomerular injury [10, 11]. We recently reported that a low glomerular density (GD) associated with GH might be a characteristic histological finding of patients with obesity-related glomerulopathy (ORG) [12]. We hypothesized that the GD, GH and obesity could be the characteristic findings of the proteinuric CKD patients without known glomerular diseases. To investigate this hypothesis, we carried out an investigation to explore the pathogenic role of GD, the glomerular volume (GV) and obesity in those patients.

## Subjects and methods

### Patient selection

**Fig. 1:**
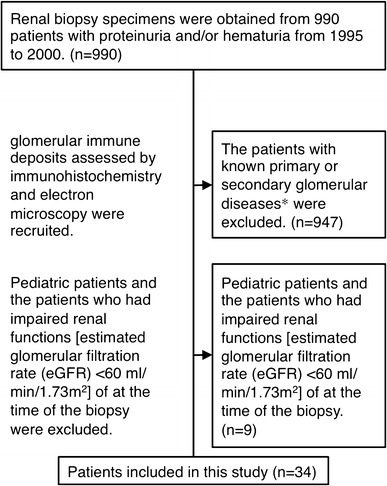
A flow diagram of patients considered for inclusion. Of the 990 Japanese patients with persistent urine abnormalities, such as proteinuria, who underwent a renal biopsy at our institute from 1995 through 2000, we excluded 947 patients with known primary or secondary glomerular diseases. Furthermore, of the remaining adult 43 cases, 9 patients having estimated glomerular filtration rate (eGFR) <60 ml/min/1.73 m^2^ at the time of the biopsy were excluded because of the probability of renal functional compensation, leaving 34 patients. * Minimal change nephrotic syndrome, FGS presenting with nephrotic syndrome and IgA nephropathy, membranous nephropathy, poststreptococcal acute glomerulonephritis, membranoproliferative nephritis, lupus nephritis, anti-glomerular basement membrane antibody nephritis, monoclonal Ig-deposition disease and other glomerulonephritis accompanied by Ig deposits, diabetic nephropathy, anti-neutrophil cytoplasmic antibody-related nephritis, amyloid nephropathy, pre-eclampsia or pregnancy-induced hypertension, thin basement membrane disease and Alport’s syndrome

Of the 990 Japanese patients who underwent a renal biopsy at our institute from 1995 through 2000, because they presented with persistent urine abnormalities, such as proteinuria, we excluded 947 patients with known primary or secondary glomerular diseases, i.e., minimal change nephrotic syndrome, focal glomerulosclerosis (FGS) presenting with nephrotic syndrome and immunoglobulin (Ig) A nephropathy, membranous nephropathy, poststreptococcal acute glomerulonephritis, membranoproliferative nephritis, lupus nephritis, anti-glomerular basement membrane antibody nephritis, monoclonal Ig-deposition disease and other glomerulonephritis accompanied by Ig deposits, diabetic nephropathy, anti-neutrophil cytoplasmic antibody-related nephritis, amyloid nephropathy, pre-eclampsia or pregnancy-induced hypertension, thin basement membrane disease and Alport’s syndrome. Furthermore, of the remaining adult 43 cases without known glomerular diseases, 9 patients having estimated glomerular filtration rate (eGFR) <60 ml/min/1.73 m^2^ at the time of the biopsy were excluded because of the probability of renal functional compensation, leaving 34 patients (Fig. 1).

### Pathological investigation

All tissue samples were collected by percutaneous needle biopsy. An 18-gauge biopsy needle was used for all biopsy cases in this study. After the tissue was embedded in paraffin, it was finely sliced into 3–4 μm sections. Hematoxylin–eosin staining, periodic acid–Schiff (PAS) staining, Masson-trichromium staining and periodic acid–methenamine silver (PAM) staining were performed. We evaluated the presence or absence of exhibiting global glomerulosclerosis, segmental glomerulosclerosis, cellular crescents, fibrocellular crescents, fibrous crescents or tuft adhesion. We also evaluated the presence or absence of an increased mesangial matrix. We semiquantified and evaluated the interstitial fibrosis and the extent of tubular atrophy according to the proportion of the total cortical area exhibiting fibrosis, and scored them as follows: 0, none; 1+, 1–25 %; 2+, 26–50 % and 3+, ≥50 %. We scored and evaluated the intimal hyalinization of the arterioles and intimal thickness of the interlobular arteries as follows: 0, no lesions; 1+, mild; 2+, moderate and 3+, severe.

The GD was determined by calculating the number of glomeruli excluding globally sclerosed glomeruli per total renal cortical area and was measured using a computed imaging analyzer (Leica IM500, Leica Microsystems, Germany). The glomerular area (GA) was defined as the area described by the outer capillary loops of the tuft using the computed imaging analyzer. The GA was measured in only one slice of the tissue section to avoid multiple measurements of the same glomeruli. The mean GA was calculated by averaging the areas of all the glomeruli. The mean glomerular volume (GV) was calculated from the measured GA according to the equation:

$${\text{GV}} = ({\text{GA}})^{3/2} \times \beta /d,$$

where *β* is a dimensionless shape coefficient (*β* = 1.38 for spheres) and *d* is a size distribution coefficient used to adjust for variations in glomerular size [13]. The analysis used *d* = 1.01, as in previous studies [14, 15].

### Definition of a hypertrophied glomerulus

We previously analyzed the renal biopsy specimens from 20 kidney transplant donors as controls [12]. Kidney transplant donors represented the healthy individuals without apparent CKD. Their mean GV ± the standard deviation (SD) was 2.4 ± 0.6 × 10^6^/μm^3^. The mean GV + 2 SD for the donors was 3.6 × 10^6^ μm^3^, which covered approximately 95 % of the donors’ GV values. Therefore, in the present study, a hypertrophied glomerulus was defined as one having a GV more than 3.6 × 10^6^ μm^3^. We separated the patients into two groups; Group 1 consisted of patients with mean GV ≥3.6 × 10^6^ μm^3^ (those with GH, *n* = 19), and Group 2 consisted of patients with mean GV <3.6 × 10^6^ μm^3^ (those without GH, *n* = 15).

### Items included in the clinical examination

The following blood parameters were measured in all patients: the levels of fasting blood glucose (FBG), serum total cholesterol (TC), triglycerides (TG), high-density lipoprotein cholesterol (HDL-C), low-density lipoprotein cholesterol (LDL-C), creatinine (Cr) and uric acid (UA). The urine parameter measured was the protein excretion over a 24-h period. The estimated glomerular filtration rate (eGFR) was calculated as follows: 194 × serum Cr level − 1.094 × age − 0.287 (female = ×0.739) [16]. To use this equation, the serum Cr levels need to be measured by an enzymatic method, which we applied in this study. The 24-h urine protein level was measured by spectrometry. The body mass index (BMI) was calculated as the weight (kg)/height (m^2^). The blood pressure was measured using a standard mercury sphygmomanometer. The mean arterial pressure (MAP) was defined as the diastolic pressure plus a third of the systolic pressure. Hypertension was defined as a systolic pressure over 140 mmHg or a diastolic pressure over 90 mmHg, or use of antihypertensive medications. The patients who were using antihypertensive medications, such as angiotensin blockers, for renoprotection despite normal blood pressure were considered to be normotensive.

### Statistical analyses

The continuous variables are expressed as the mean ± SD. The variables were assessed for normality both visually (normal probability plot) and by inferential statistics (Kolmogorov–Smirnov tests and Bartlett test). The clinical findings at the time of the biopsies for Group 1 and Group 2 were compared using Student’s *t* test and Fisher’s exact probability test, and the pathological findings were compared using Fisher’s exact probability test and the Mann–Whitney *U* test. Non-parametric variables were expressed as medians and interquartile ranges (IQR) and were compared using the Mann–Whitney *U* test. Next, we examined the correlations between the individual mean GV and the clinical or pathological findings at the time of biopsy for all 34 cases, using the univariate regression analysis and the stepwise multivariate regression analysis. The factors associated with the mean GV in the univariate regression analysis were selected for inclusion as the independent valuables in the stepwise multivariate regression analysis. We further analyzed these CKD patients’ kidney tissues to investigate the effects of obesity on the GD and GV. We compared the clinical and pathological variables among three groups categorized according to the BMI: non-obese (BMI <25 kg/m^2^), overweight (25 < BMI ≤ 30 kg/m^2^) and obese (BMI ≥30 kg/m^2^). The Kruskal–Wallis test, the one factor analysis of variance (ANOVA) and the Chi squared test were applied for comparisons of the variations among these three categories, and the Tukey–Kramer method was used for multiple comparisons among them. The StatView software program (SAS Institute Inc., Cary, NC, USA), version 5.0, was used for all of the analyses.

## Results

### Comparison of the clinical and pathological findings at biopsy between groups 1 and 2

As shown in Table 1, Group 1 had significantly higher values for the proportion of males and hypertensive patients, the BMI, MAP, TC, TG, Cr and UA, and significantly lower values for HDL-C. No significant difference was found in the daily urine protein excretion between the two groups. In comparison with Group 2, the patients in Group 1 had significantly higher values for the number of patients with globally sclerosed glomeruli and for the score of patients with arteriolar hyalinosis, and significantly lower values for GD (Table 2).

**Table 1:** Clinical characteristics of patients with and without glomerular hypertrophy at the time of the renal biopsy

	Group 1: patients with glomerular hypertrophy (*n* = 19)	Group 2: patients without glomerular hypertrophy (*n* = 15)	*p* value
Male (%)	94	40	0.002^a^
Age (years)	42 ± 9	42 ± 18	0.995^b^
BMI (kg/m^2^)	27 ± 3	22 ± 4	<0.001^b^
MAP (mmHg)	102 ± 12	87 ± 10	<0.001^b^
Hypertension (%)	58	20	0.038^a^
TC (mg/dl)	237 ± 59	196 ± 49	0.036^b^
TG (mg/dl)	216 ± 102	132 ± 90	0.018^b^
HDL-C (mg/dl)	46 ± 12	55 ± 10	0.045^b^
FBG (mg/dl)	96 ± 13	88 ± 22	0.269^b^
Cr (mg/dl)	0.8 ± 0.2	0.6 ± 0.2	0.046^b^
eGFR (ml/min/1.73 m^2^)	86.5 (74.5, 101.9)	100.2 (89.1, 121.8)	0.086^c^
UA (mg/dl)	7.3 ± 1.5	5.3 ± 1.5	<0.001^b^
Urinary protein excretion rate (g/day)	0.70 (0.40, 1.04)	0.41 (0.36, 0.61)	0.182^c^

Values are expressed as the percentage of patients, mean ± SD or medians [interquartile ranges (IQR)]

*BMI* body mass index, *MAP* mean arterial pressure, *TC* total cholesterol, *TG* triglycerides, *HDL-C* high-density lipoprotein cholesterol, *FBG* fasting blood glucose, *Cr* creatinine, *eGFR* estimated glomerular filtration rate, *UA* uric acid

^a^Fisher’s exact probability test

^b^Student’s *t* test (mean ± SD)

^c^Mann–Whitney *U* test [median (IQR)]

**Table 2:** Renal histological findings of patients with and without glomerular hypertrophy

	Group 1: patients with glomerular hypertrophy (*n* = 19)	Group 2: patients without glomerular hypertrophy (*n* = 15)	*p* value
Patients with globally sclerosed glomeruli	14	3	0.005^a^
Patients with segmentally sclerosed glomeruli	3	1	0.613^a^
Patients with increased mesangial matrix	3 focal segmental in 2 patients	1 focal segmental in a patient	>0.999^a^
Score of patients with interstitial fibrosis	1(+) in 18 patients 2(+) in 1 patients	1(+) in 10 patients	0.060^b^
Score of patients with arteriolar hyalinosis	1(+) in 6 patients 2(+) in 8 patients 3(+) in 4 patients	1(+) in 3 patients 2(+) in 1 patients 3(+) in 2 patients	0.036^b^
Score of patients with increased arterial fibrous intimal thickness	1(+) in 6 patients 2(+) in 3 patients	1(+) in 3 patients 2(+) in 2 patient	0.392^b^
GD	2.0 ± 0.7	3.3 ± 1.2	<0.001^c^

Values are expressed as the number of patients or mean ± SD

*GD* glomerular density excluding global glomerular sclerosis

^a^Fisher’s exact probability test

^b^Mann–Whitney *U* test

^c^Student’s *t* test

### Clinical and pathological findings associated with the mean GV

In the univariate regression analysis, the individual mean GV was significantly associated with the BMI, sex, MAP, Cr and UA at the time of the renal biopsy (Table 3). Concerning the pathological parameters, the mean GV was significantly associated with GD, as well as the degrees of globally sclerosed glomeruli, interstitial fibrosis and arteriolar hyalinosis. The stepwise multiple linear regression analyses were performed using the BMI, sex, MAP, Cr, UA, GD, and the degrees of globally sclerosed glomeruli, interstitial fibrosis and arteriolar hyalinosis, as independent variables. The analyses revealed that the BMI, sex and GD were significant factors correlated with the mean GV.

**Table 3:** Clinical and pathological findings associated with mean GV (univariate regression model and multivariate stepwise regression model) (*n* = 34)

	Univariate		Multivariate (stepwise)	
	*r*	*p* value	*β*	*p* value
Sex	0.613	0.0001	0.371	<0.0001
BMI	0.638	<0.0001	0.366	<0.0001
MAP	0.436	0.0100	–	–
TC	0.196	0.2661		
TG	0.248	0.1575		
HDL-C	−0.313	0.0861		
FBG	0.156	0.4367		
Cr	0.426	0.0120	–	–
eGFR	−0.146	0.4089		
UA	0.495	0.0047	–	–
Urine protein excretion rate	0.054	0.7627		
Degree of globally sclerosed glomeruli	0.364	0.0344	–	–
Degree of segmentally sclerosed glomeruli	0.020	0.9085		
Degree of interstitial fibrosis	0.570	0.0004	–	–
Degree of arteriolar hyalinosis	0.430	0.0112	–	–
Degree of arterial fibrous intimal thickness	0.212	0.2373		
GD	−0.581	0.0003	−0.289	<0.0001

*BMI* body mass index, *MAP* the mean arterial pressure, *TC* total cholesterol, *TG* triglyceride, *HDL-C* high-density lipoprotein cholesterol, *FBG* levels of fasting blood glucose, *Cr* creatinine, *eGFR* the estimated glomerular filtration rate, *UA* uric acid, *GD* glomerular density excluding global glomerular sclerosis

### Comparison of the different BMI categories

As shown in Table 4, the values for GD, as well as those for the eGFR, were significantly different among the non-obese, overweight and obese groups. The values for the mean GV were also significantly different among these three groups. The values for the mean GV were significantly higher in the overweight and obese groups than in the non-obese group, and the values for GD were significantly lower in the obese group than in the non-obese group.

**Table 4:** Clinical and histological findings of the patients categorized by body mass index

Characteristics	Non-obese (*n* = 13)	Overweight (*n* = 18)	Obese (*n* = 3)	*p* value
Clinical				
Age (years)	38 (29, 49)	41 (37, 46)	50 (41, 54)	0.479^a^
Male (%)	46	80	100	0.066^c^
eGFR (ml/min/1.73 m^2^)	110 ± 26	91 ± 20	71 ± 9^†^	0.015^b^
Histopathologic				
GD (glomeruli/μm^2^)	3.3 ± 1.2	2.2 ± 1.0	1.8 ± 0.6^†^	0.021^b^
Mean GV (×10^6^/μm^3^)	2.4 ± 1.3	3.6 ± 0.9^†^	4.7 ± 0.8^†^	0.026^b^

Values are expressed as the percentage of patients, mean ± SD or median [interquartile ranges (IQR)]

*BMI* body mass index, *eGFR* the estimated glomerular filtration rate, *GD* glomerular density excluding global glomerular sclerosis, *mean GV* mean glomerular volume

^†^ *p* < 0.05 vs. non-obese by multiple comparisons using the Tukey–Kramer method

^a^The Kruskal–Wallis test

^b^The one factor analysis of variance (ANOVA) test

^c^Chi square test

## Discussion

Our major goal was to clarify the pathogenic role of the GD, GV and obesity in proteinuric CKD patients without known glomerular diseases. When our 34 patients were divided into two groups based on the presence or absence of a mean GV which fulfilled the definition of GH (GV >3.6 × 10^6^ μm^3^), the patients with GH (Group 1) showed significantly higher values for the BMI, MAP and UA, and a significantly higher frequency of male patients compared to those without GH (Group 2). Of note, the patients in Group 1 had significantly lower GD values as compared to Group 2 patients, whereas the degrees of other pathological changes were comparable between the two groups, except for the score of patients with arteriolar hyalinosis and the frequency of patients with global sclerosed glomeruli (Table 2). The stepwise multivariate regression analyses for all 34 patients revealed that the GD, sex and BMI were independent factors significantly associated with the mean GV (Table 3).

Among the three subgroups of patients categorized according to the BMI, i.e., non-obese (BMI <25 kg/m^2^), overweight (25 < BMI ≤ 30 kg/m^2^) and obese (BMI ≥30 kg/m^2^) patients, the GD values, as well as the eGFR, were significantly lower in the groups with higher BMI values. Thus, among our proteinuric CKD patients, we could find a subgroup of patients who had higher BMI and GV values and a lower GD, as common clinical and pathological features.

There have been various investigations into the relationship between obesity and renal impairment [17, 18]. Kambham et al. [19] defined a new entity, ORG, in which GH with FGS lesions or only GH developed in obese patients with a BMI of 30 kg/m^2^ or more, and proposed ORG as a renal disease that has been increasing in prevalence in recent years. These previous studies examined the renal histological features of obese patients with a BMI of 30 kg/m^2^ or more. In contrast, the present study examined the characteristics of proteinuric patients without known primary or secondary glomerular diseases, especially focusing on the glomerular volume in the kidney biopsy specimens. We found that higher BMI levels, even if they were <30 kg/m^2^, had a significant correlation with the enlargement of the GV. Therefore, the present study was unique in terms of the methodology, which was based on the glomerular volume, not the BMI.

We recently reported that a low GD associated with GH may be a characteristic histological finding of patients with ORG [12]. In that study, the analysis of autopsy cases without CKD, which were characterized by having an eGFR ≥60 ml/min/1.73 m^2^ and no persistent urinary abnormalities, showed that the GD in overweight or obese persons was similar to that in non-obese individuals, although the GV was larger in the overweight and obese groups as compared to the non-obese group, among the autopsy cases. In contrast to those results, we found in the present study that the GD levels in our proteinuric patients were significantly lower in the obese group as compared to the non-obese group. In addition, the GD had a significant inverse correlation with the GV in our 34 patients (Table 3), indicating the functional adaptation of remaining glomeruli in patients with a small number of functioning nephrons. Based on these findings, it is plausible to speculate that, in the patients with a low GD and large GV, obesity-related hemodynamic changes such as an increase of plasma flow or blood pressure within the glomerulus can alter glomerular permselectivity. Thus, a low GD may play a crucial role in the development of proteinuria in association with GH in overweight or obese persons.

Concerning the pathological findings of our 34 proteinuric patients, the population of patients with increased mesangial matrix was comparable between those with and without GH (Table 2), indicating that GH was caused by the enlargement of glomerular capillaries. Sasatomi et al. [20] previously demonstrated, using glomerular morphometry, that the GH observed in obese patients presenting with urine abnormalities was due to the enlargement of glomerular capillaries. This finding was consistent with our results showing that there was no significant mesangial matrix increase in the hypertrophied glomeruli.

There are several limitations of this study that should be kept in mind when interpreting the results. First, it is uncertain whether the GD on a renal biopsy specimen represents the total nephron number of the whole kidney. Therefore, the finding of a low GD observed in the patients with GH may not necessarily reflect a low number of glomeruli. Accurately determining the origin of the low GD in the biopsy specimens of those with GH requires further investigations. Second, there is a possibility that some of the 34 patients might have had FGS without nephrotic syndrome or benign nephrosclerosis, because these two diseases could not be completely excluded merely on the basis of the morphological findings. However, the possibility of the presence of FGS patients would be considerably low, since only four patients (12 %) had segmentally sclerosed glomeruli in this study. Some patients with benign nephrosclerosis may also have been enrolled in this study, since most of the patients with GH had arteriolar hyalinosis. Nevertheless, it was meaningful that the subpopulation of patients with benign nephrosclerosis could be identified by the characteristics of low GD with GH on the biopsy specimens, if such cases had been included in our study.

In summary, among the 34 proteinuric CKD patients without known glomerular diseases, those with GH had significantly lower GD compared to those without GH. The BMI and GD values were identified as significant factors that correlated with the mean GV. The values for the mean GV were significantly higher in the overweight and obese groups than in the non-obese group, and the values for the GD were significantly lower in the obese group than in the non-obese group. Thus, we could identify a subgroup of patients who were characterized as having a high BMI and GV and a low GD, among the proteinuric CKD patients without known glomerular diseases.
